# Stable symmetry-protected 3D embedded solitons in Bose–Einstein condensates

**DOI:** 10.1038/s41598-018-29219-7

**Published:** 2018-07-19

**Authors:** V. Delgado, A. Muñoz Mateo

**Affiliations:** 1 Departamento de Física, Facultad de Ciencias, Universidad de La Laguna, La Laguna, Tenerife, Spain; 2 Dodd-Walls Centre for Photonic and Quantum Technologies and Centre for Theoretical Chemistry and Physics, New Zealand Institute for Advanced Study, Massey University, Auckland, 0745 New Zealand

## Abstract

Embedded solitons are rare self-localized nonlinear structures that, counterintuitively, survive inside a continuous background of resonant states. While this topic has been widely studied in nonlinear optics, it has received almost no attention in the field of Bose–Einstein condensation. In this work, we consider experimentally realizable Bose–Einstein condensates loaded in one-dimensional optical lattices and demonstrate that they support continuous families of stable three-dimensional (3D) embedded solitons. These solitons can exist inside the resonant continuous Bloch bands because they are protected by symmetry. The analysis of the Bogoliubov excitation spectrum as well as the long-term evolution after random perturbations proves the robustness of these nonlinear structures against any weak perturbation. This may open up a way for the experimental realization of stable 3D matter-wave embedded solitons as well as for monitoring the gap-soliton to embedded-soliton transition.

## Introduction

Solitons have attracted much interest since their discovery in 1834. They are a peculiar manifestation of nonlinear wave systems resulting from a detailed balance between dispersion and nonlinearity^1^. Solitons are ubiquitous in Physics, where they appear as particle-like self-localized coherent solutions of certain differential equations such as the well-known nonlinear Schrödinger equation. This equation has great relevance in the field of nonlinear optics, where it governs the propagation of the envelope of an electromagnetic pulse^1^ and plays also an essential role in the field of Bose–Einstein condensation^2^, where it adopts the form of the Gross–Pitaevskii equation (GPE) and describes the dynamics of Bose–Einstein condensates (BECs) of dilute atomic gases^3,4^. Not surprisingly, in both the above fields the study of solitons have received great attention in recent years. In nonlinear optics, solitons have found broad practical application in telecommunications via optical fibers. In Bose–Einstein condensation, dark solitons have been experimentally realized in atomic condensates with repulsive interparticle interactions^5,6^. These solitons represent the matter-wave counterpart of optical dark solitons in self-defocusing nonlinear media. Matter-wave bright solitons have been obtained in attractive condensates (the equivalent of self-focusing nonlinear media)^7,8^. The matter-wave analogue of optical gap solitons has also been experimentally observed in repulsive condensates of ^87^Rb atomic gases^9^. Matter-wave gap solitons are self-trapped nonlinear structures that can be found in BECs immersed in the periodic potential of an optical lattice^10,11^. They are localized nonlinear stationary states of the GPE whose chemical potentials lie in the forbidden band gaps of the linear spectrum. In a repulsive condensate, gap soliton solutions bifurcate from the upper edge of the linear Bloch bands, forming distinct one-parameter families characterized by their chemical potential *μ* as a function of the number of atoms *N*^12,13^. These families describe continuous trajectories in the *μ* − *N* parameter plane and, in general, vanish as they approach the vicinity of an upper band, where the gap solitons become resonant with the extended Bloch waves residing therein. In these regions, in general, only delocalized solutions with nonvanishing oscillating tails can exist. However, under certain circumstances localized solitons can be found inside the continuous background of resonant states^14^. These solitons, that reside in the continuous spectrum, were called embedded solitons and studied in ref.^15^ in the framework of nonlinear optics. Both isolated (at specific parameter values) and continuous families of embedded solitons have been reported in the literature and, usually, they were found to be semistable^14–17^. Most studies have been restricted to the one-dimensional case and finding higher dimensional embedded solitons still remains a challenge. In this regard, continuous families of fully stable 2D embedded solitons were obtained in ref.^18^ in a self-defocusing optical medium in a quasi-1D waveguide array characterized by a refractive index variation

1 $$n(x,y)=6\,{e}^{-{y}^{2}/4}{\cos }^{2}x.$$

Symmetry plays an important role in the existence of these solitons, which can only exist inside Bloch bands with opposite parity from the solitons themselves^18^.

Embedded solitons have received almost no attention in the field of Bose–Einstein condensation. This may be, in part, because most theoretical studies on gap solitons have focused on quasi-1D BECs^10,12,13,19^. Nonetheless, 3D gap waves^20^ (a type of gap solitons that can be viewed as truncated nonlinear Bloch waves) and 3D gap solitons^21,22^ have been obtained, respectively, in BECs loaded in 3D and in 1D optical lattices. On the other hand, the authors of ref.^23^ have studied embedded solitons using a generalized 2D GPE that incorporates an extra momentum operator of the form −*i*(*γ*_*x*_∂_*x*_ + *γ*_*y*_∂_*y*_), where *γ*_*x*_ and *γ*_*y*_ are adjustable parameters. They found that in the presence of a periodic potential *V*(*x*, *y*) of the same functional form as the refractive index *n*(*x*, *y*) of Eq. (1), this theoretical model admits stable 2D embedded solitons which, as occurs with those previously found in ref.^18^, can only exist inside Bloch bands with opposite symmetry to that of the solitons.

Despite all the efforts, no stable 3D embedded solitons have been obtained so far neither in nonlinear optics nor in BECs. In this work, we obtain for the first time stable 3D embedded solitons. Specifically, we consider experimentally realizable single-component BECs subject to a sufficiently weak transverse confinement and loaded in 1D optical lattices and demonstrate that they support *continuous* families of *stable* 3D gap-solitons that can survive inside the continuous background of resonant Bloch waves. Since, under these circumstances, the GPE commonly does not admit localized stationary solutions, the existence of such embedded solitons requires the fulfillment of certain special conditions. The question arises as to why these embedded solitons can exist. To address this question, we perform a realistic 3D numerical treatment that enables us to follow the gap-soliton to embedded-soliton transition and to understand the physics behind their existence. Our numerical results indicate that these solitons can exist inside the resonant Bloch bands because they are protected by rotational symmetry. By calculating the Bogoliubov excitation spectrum and the long-term nonlinear evolution after random perturbations, we have checked that these solitons are fully stable, which may open up a way for experimentally generating robust 3D embedded solitons in BECs and for investigating the corresponding gap-soliton to embedded-soliton transition.

## Model

In this work, we consider a ^87^Rb condensate subject to a transverse confinement of frequency *ω*_⊥_/2*π* = 320 Hz and loaded in a 1D optical lattice of period *d* = *π*/2 *μ*m and depth *s* = 15 in units of the recoil energy *E*_*R*_ ≡ ℏ^2^*π*^2^/2*m*_0_*d*^2^ = 0.75ℏ*ω*_⊥_ (where *m*_0_ is the atomic mass). Its dynamics is governed by the 3D GPE

2 $$i\hslash {\partial }_{t}\psi =(-{\hslash }^{2}{\nabla }^{2}/2{m}_{0}+V({\bf{r}})+gN{|\psi |}^{2})\psi ,$$

which accurately describes the physics of zero-temperature BECs in the mean field regime under realistic experimental conditions^24,25^. In this equation, $V({\bf{r}})=\frac{1}{2}{m}_{0}{\omega }_{\perp }^{2}{{\bf{r}}}_{\perp }^{2}+s{E}_{R}{\sin }^{2}(\pi z/d)$ is the external potential, *N* is the number of atoms in the condensate, and *g* = 4*πℏ*^2^*a*/*m*_0_ is the interatomic interaction strength, with *a* = 5.29 nm being the s-wave scattering length. To determine the spectrum of the underlying linear problem, we note that the Hamiltonian is separable. The transverse eigenvalue equation can be solved analytically. Its solutions are the eigenmodes of the radial harmonic oscillator

3 $${\phi }_{{n}_{r}}^{(m)}(\rho ,\theta )=\sqrt{\frac{{n}_{r}!}{\pi {a}_{\perp }^{2}({n}_{r}+|m|)!}}{e}^{im\theta }{\rho }^{|m|}{e}^{-{\rho }^{2}\mathrm{/2}}{L}_{{n}_{r}}^{(|m|)}({\rho }^{2}),$$

with corresponding eigenvalues

4 $${E}_{m,{n}_{r}}=\mathrm{(2}{n}_{r}+|m|+\mathrm{1)}\hslash {\omega }_{\perp }.$$

${L}_{{n}_{r}}^{(|m|)}({\rho }^{2})$ are generalized Laguerre polynomials, with *ρ* ≡ *r*_⊥_/*a*_⊥_ where ${a}_{\perp }=\sqrt{\hslash /{m}_{0}{\omega }_{\perp }}$ is the harmonic-oscillator length and (*r*_⊥_, *θ*) are the polar coordinates; *m* = 0, ±1, ±2, … is the axial angular momentum quantum number, and *n*_*r*_ = 0, 1, 2, … is the radial quantum number. Thus, the problem reduces to solving the 1D axial eigenvalue equation. This is a stationary Schrödinger equation in a periodic potential whose solutions are Bloch waves *ϕ*_*n*,*q*_(*z*) = *e*^*iqz*^*u*_*n*_(*z*), characterized by the band index *n* and the quasimomentum *q*. The resulting 3D spectrum reproduces the band-gap structure of the 1D axial problem for every (*m*, *n*_*r*_) transversal state and consists of a series of 3D Bloch bands determined by the quantum numbers (*n*, *m*, *n*_*r*_).

**Figure 1:**
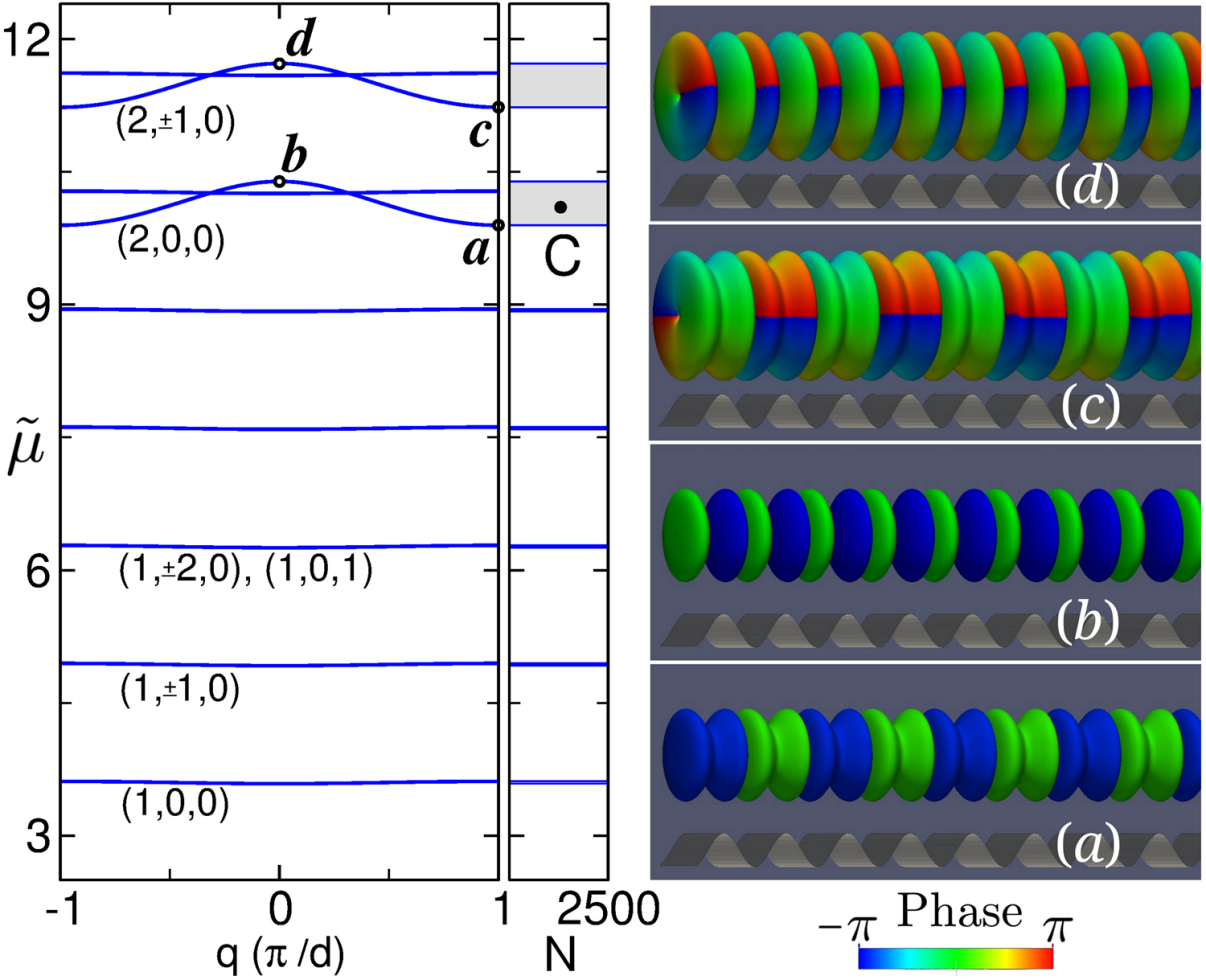
Left: Ideal-gas (*gN* = 0) linear spectrum. Point C corresponds to an embedded soliton with *N* = 1130. Right: Phase-colored density isosurfaces of the Bloch waves corresponding to the points labeled with the same letters on the left.

This spectrum is depicted in Fig. 1, which shows the scaled chemical potential $\tilde{\mu }\equiv (\mu -\hslash {\omega }_{\perp })/{E}_{R}$ as a function of *q*. For a given band index *n*, there exists an infinite series of replicas of the lowest-energy Bloch band (*n*, 0, 0) corresponding to the different (*m*, *n*_*r*_) excited transversal states^12,21,22^. Panels (*a*)–(*d*) in Fig. 1, show the Bloch waves corresponding to the points labeled with the same letters on the left, represented as phase-colored isosurfaces of the atom density taken at 5% of its maximum. The optical lattice is also shown for reference at the bottom. Panels (*a*) and (*b*) correspond to the minimum and maximum chemical potential of the (2, 0, 0) band, while panels (*c*) and (*d*) correspond to the equivalent points of the (2, 1, 0) band. Both of these bands originate from the first excited Bloch band of the 1D axial problem. Bloch waves in this excited 1D band consist of two out-of-phase axial peaks (one axial node) at every lattice site. The main difference between the eigenmodes displayed in the figure is that while Bloch modes in the (2, 0, 0) band are in their (topologically trivial) transverse ground state, those in the (2, 1, 0) band exhibit a central vortex with topological charge *m* = 1.

Because of the axial symmetry of the Hamiltonian *H*, eigenstates with different values of |*m*| (which belong to different irreducible invariant subspaces of the rotation group) cannot be dynamically connected by *H*. As we will see, this fact remains true in the nonlinear regime and ultimately allows for the existence of symmetry-protected embedded solitons inside the resonant Bloch bands. Point *C* in Fig. 1 marks the location of one of these embedded solitons with *N* = 1130.

**Figure 2:**
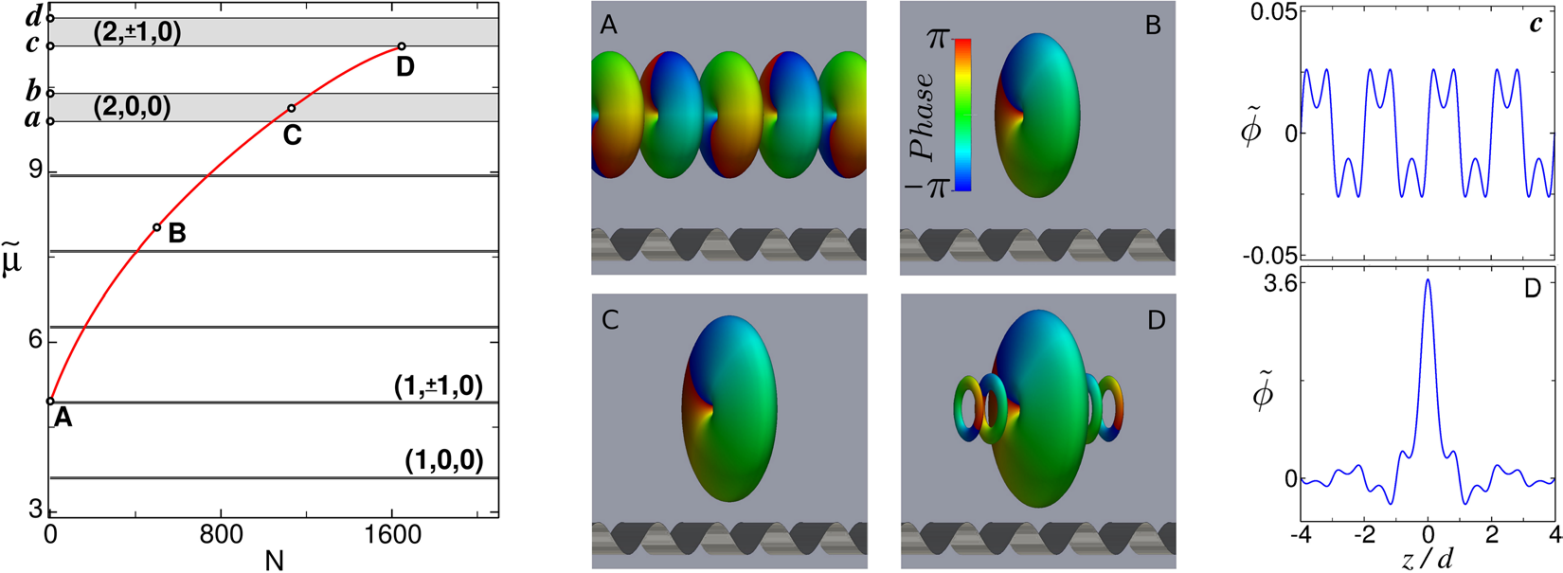
Left: Trajectory in *μ* − *N* plane of the (1, 1, 0) soliton family. Middle: Phase-colored density isosurfaces corresponding to the points labeled with the same letters on the left panel. Right: Axial profiles (in arbitrary units) of the 3D wave functions corresponding to the points *c* and *D* on the leftmost panel. Note that panel *c* refers to the wave function shown in Fig. 1(c) while panel D refers to the wave function shown in the middle panel D of the present figure.

While in the linear regime all the stationary states of the GPE in an optical lattice are extended (delocalized) Bloch waves, in the nonlinear regime there also exist stationary solutions in the form of families of self-localized gap solitons residing within the band gaps. To obtain these gap soliton families, we numerically seek stationary solutions of the form *ψ*(***r***, *t*) = *ψ*_0_(***r***)exp(−*iμt*/*ℏ*), where the chemical potential *μ* is used as the continuation parameter for a Newton continuation method implemented in terms of a Laguerre–Fourier spectral basis consisting of the eigenfunctions ${\phi }_{{n}_{r}}^{(m)}(\rho ,\theta )$ of the radial harmonic oscillator, Eq. (3), along with the plane-wave eigenfunctions of the axial linear momentum *P*_*z*_ = −*iℏ*∂_*z*_. As the continuation parameter *μ* increases (or equivalently, as *N* increases), gap soliton families describe distinctive trajectories *μ*(*N*) in the *μ* − *N* plane. Figure 2 displays the trajectory characterizing the family of fundamental gap solitons of type (1, 1, 0), where, following the notation introduced in refs^12,21,22^, we are denoting gap soliton families by the quantum numbers of the linear Bloch band from which they bifurcate. The middle panels show the 3D stationary wave functions of points *A*–*D* in terms of phase-colored isosurfaces of the atom density, while the right panels depict the axial profiles (obtained by integrating out the radial coordinate for a fixed *θ*) of the 3D wave functions of points *c* and *D* (see Figs 1(c) and 2(D), respectively). In the linear regime (point *A*), the stationary solution in this family is an extended Bloch wave featuring an axisymmetric vortex with topological charge *m* = 1 (panel *A*). Note the *π*-phase shift along the *z* axis for every given *θ* in addition to the 2*π*-phase shift around the vortex singularity. As already said, in the nonlinear regime (*gN* > 0), localized stationary solutions appear inside the band gaps of the linear spectrum. These solutions, which bifurcate from the upper edge of the Bloch band giving rise to the continuous *μ*(*N*) family shown in the figure, become more localized as one moves away from the Bloch band. A representative example of a gap soliton in this family (point *B*) is shown in Panel *B*. As can be seen, it is a gap soliton with topological charge *m* = 1, fully localized inside a single potential well. All solitons in this family are eigenstates of the axial angular momentum *L*_*z*_ with eigenvalue *m* = 1.

Most commonly, characteristic trajectories *μ*(*N*) of gap soliton families vanish as they enter Bloch bands. This occurs because inside the allowed energy bands, gap solitons resonantly couple with the extended linear Bloch waves, which eventually prevents the existence of localized stationary states. However, as can be seen in Fig. 2, gap solitons in this family survive inside the (2, 0, 0) Bloch band. These surviving solitons, with chemical potential $\tilde{\mu }$ between points *a* and *b*, represent a continous *μ*(*N*) family of symmetry-protected 3D embedded solitons. Point *C* in Figs 1 and 2 constitutes a representative example of one of these solitons. As can be seen in panel *C*, this soliton is a genuine self-localized 3D embedded soliton with topological charge *m* = 1. Its existence is a direct consequence of the rotational symmetry of the system, which forbids the solitons in this family |*ψ*_*m*_〉 to couple with resonant linear Bloch waves |*ϕ*_*m*′_〉 with different values of *m*. Indeed, taking into account that |*ψ*_*m*_〉 are eigenstates of the nonlinear hamiltonian, *H*|*ψ*_*m*_〉 = *μ*|*ψ*_*m*_〉, and that both |*ψ*_*m*_〉 and |*ϕ*_*m*′_〉 are eigenstates of *L*_*z*_, one finds 〈*ϕ*_*m*′_|*H*|*ψ*_*m*_〉 = *μ*〈*ϕ*_*m*′_|*ψ*_*m*_〉 ∝ *δ*_*m*′*m*_. Thus, *H* cannot dynamically couple soliton families with Bloch waves having different rotational properties. They are protected by symmetry. As a result, fundamental solitons of the (1, 1, 0) family can safely reside within the (2, 0, 0) Bloch band. On the contrary, as is apparent from the figure, no localized (1, 1, 0) soliton solutions can be found inside the (2, 1, 0) spectral band. In fact, as gap solitons in this family approach the lower edge of the Bloch band (point *D*), they develop an oscillating tail reminiscent of the Bloch waves residing in the spectral band. This can be seen from the middle panel *D* in Fig. 2, that shows the density isosurface of the gap soliton corresponding to point *D*. As is apparent, in this case the gap soliton appears flanked on each side by a density pattern that exhibits the characteristic features of the Bloch waves displayed in Fig. 1(c) (two out-of-phase axial peaks (one axial node) at each lattice well and a unit-charge central vortex). Since in isosurface plots the atom density is abruptly cut off, it can be more illustrative to consider the two rightmost panels *c* and *D* of Fig. 2, which depict, respectively, the axial profiles of the 3D wave functions shown in Fig. 1(c) and in the middle panel *D* of Fig. 2. A simple comparison reflects that in this case, in the vicinity of the Bloch band, gap solitons become contaminated by the extended Bloch waves residing therein.

**Figure 3:**
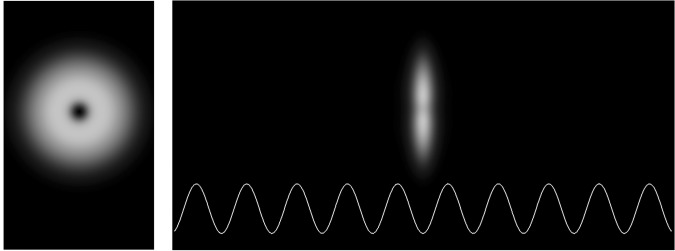
Column densities of the embedded soliton *C* of Fig. 2 integrated along the axial (left panel) and transverse (right panel) directions.

In Fig. 3 we show the axial and transverse column densities of the embedded soliton *C* of Fig. 2, which are the quantities directly measured in experiments.

We have also investigated the family of fundamental (1, 0, 0) gap solitons. Besides bifurcating from the lowest Bloch band and being, consequently, in their topologically trivial ground state, the numerical results obtained for the solitons in this family are qualitatively similar to those of Fig. 2 and are not explicitly shown. As expected, solitons in this family survive protected by symmetry inside the (2, ±1, 0) Bloch band, while they do not exist in the vicinity of the (2, 0, 0) band. As we will see, however, these (1, 0, 0) embedded solitons have different stability properties than the topologically protected (1, 1, 0) solitons considered above.

It is worth remarking the crucial role that the dimensionality of the problem plays in the existence and properties of the embedded solitons considered in this work. While certain properties of gap solitons, such as the chemical potential *μ*(*N*), can be obtained in an approximate way in terms of an effectively 1D model, the very existence of the above embedded solitons is a direct consequence of a specific interplay between axial and transverse degrees of freedom, which clearly cannot be accounted for by any 1D model. This is a genuine 3D system in which transverse and axial degrees of freedom play an equally relevant role that must be explicitly incorporated in the description of the problem.

## Stability

Dynamical stability is a necessary prerequisite for the above embedded solitons to have any physical relevance. To investigate this issue, we begin by performing a linear stability analysis based on the Bogoliubov spectrum of elementary excitations. To this end, we perturb the stationary wavefunctions *ψ*_0_(**r**) of the embedded solitons by introducing a small fluctuation of frequency *ω*

5 $$\psi ({\bf{r}},t)=[{\psi }_{0}({\bf{r}})+u({\bf{r}}){e}^{-i\omega t}+{v}^{\ast }({\bf{r}}){e}^{i\omega t}]{e}^{-i\mu t/\hslash }.$$

**Figure 4:**
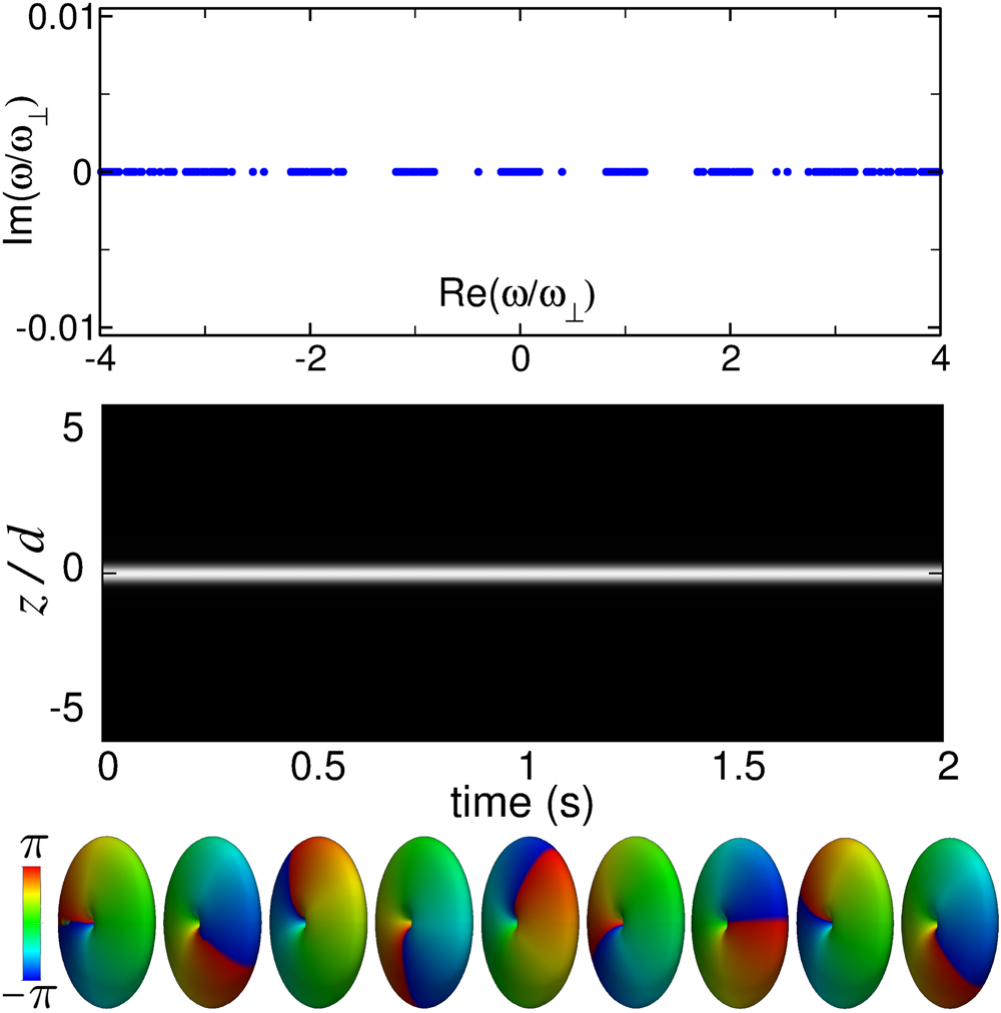
Results of the stability analysis of the embedded soliton *C* of Fig. 2.

After substituting in the GPE and retaining up to linear terms in the amplitudes *u* and *v*, one obtains the corresponding Bogoliubov–de Gennes (BdG) equations

6 $$(\begin{array}{cc} {\mathcal L}  & gN{\psi }_{0}^{2}({\bf{r}})\\ -gN{\psi }_{0}^{\ast 2}({\bf{r}}{\boldsymbol{)}} & - {\mathcal L} \end{array})(\begin{array}{c}u({\bf{r}})\\ v({\bf{r}})\end{array})=\hslash \omega (\begin{array}{c}u({\bf{r}})\\ v({\bf{r}})\end{array}),$$

where ${\mathcal L} =-\,({\hslash }^{2}\mathrm{/2}{m}_{0}){\nabla }^{2}+V({\bf{r}})-\mu +2gN{|{\psi }_{0}|}^{2}$. The solution of the above linear eigenvalue problem provides the desired information. We have numerically solved Eq. (6) by expanding its eigenfunctions in terms of the above Laguerre–Fourier spectral basis. Figure 4 collects the results of the stability analysis of the embedded soliton C of Fig. 2. As can be seen in the upper panel, that shows the spectrum of elementary excitations, all the BdG eigenfrequencies are real, which demonstrates the linear stability of this soliton. To investigate the stability in the nonlinear regime, we have produced a random perturbation in the stationary wavefunction of the soliton by adding a small-amplitude (~1%) Gaussian white noise and have analyzed its subsequent nonlinear evolution. To this end, we have numerically integrated the 3D GPE by using a pseudospectral method based on the above Laguerre–Fourier spectral basis along with a third-order Adams–Bashforth scheme for the time evolution. The middle panel in Fig. 4 shows the long-time behavior (up to *t* = 2 s) of the axial profile of the perturbed soliton by means of a density map where bright pixels indicate high densities. Below this panel, and using the same time axis, the corresponding 3D wave functions are displayed as phase-colored density isosurfaces. As is apparent, apart from the expected stationary-state global phase evolution, the embedded soliton remains unaltered, which demonstrates its stability.

It is interesting to note that stable (1, 1, 0) embedded solitons can also be found in the narrow (1, *m*, *n*_*r*_) Bloch bands with |*m*| ≠ 1, while they disappear inside the |*m*| = 1 resonant bands. In general, gap and embedded solitons in this (1, 1, 0) family are stable along the entire *μ*(*N*) trajectory except in the close proximity to the |*m*| = 1 resonant bands where they become unstable and eventually disappear as they enter the band. The soliton *D* of Fig. 2 is a representative example of one of these unstable gap solitons.

On the other hand, while the symmetry-protected (1, 0, 0) solitons embedded in the (2, ±1, 0) Bloch band are linearly stable against weak perturbations that respect the rotational symmetry of the Hamiltonian, they are not, however, fully stable. Indeed, the analysis of the BdG spectrum reveals that all the rotationally symmetric elementary excitations have real eigenfrequencies, while there exist nonsymmetric elementary excitations with complex eigenfrequencies. Moreover, their long-term nonlinear behavior after random perturbations shows that they are semistable: They remain stable under energy-increasing perturbations but decay under energy-decreasing perturbations. This is in contrast with the results obtained above for the topologically protected (1,1,0) embedded solitons. In this case, the existence of an integer-quantized topological charge makes the solitons fully stable and thus particularly amenable to experimental observation.

We propose the experimental realization of these (1, 1, 0) embedded solitons through a three-stage procedure along the lines of ref.^9^, by using a crossed dipole trap configuration. As a first stage, a (dynamically stable) single charged vortex has to be nucleated in the atomic cloud, and to this end a 2-photon stimulated Raman process with a Laguerre-Gaussian beam along the lattice (to be ramped later) could provide the corresponding quantum ℏ of orbital angular momentum per atom^26^. Next, following ref.^9^, the lattice can be adiabatically ramped up and the atomic cloud released on the associated waveguide. Finally, in order for the system to reach the boundary of the Brillouin zone from which the gap solitons originate, the lattice has to be boosted up to a quasimomentum value of *π*/*d*. By choosing the proper number of atoms for the system parameters, the resulting vortex state could be found either in a band gap (as a gap soliton) or in an energy band with zero angular momentum, thus realizing an embedded soliton.

## Conclusion

In conclusion, we have (numerically) obtained for the first time stable 3D embedded solitons. In particular, we have shown that single-component BECs in 1D optical lattices support continuous families of stable symmetry-protected 3D embedded solitons in a region of the parameter space that is readily accessible with current experimental capabilities. By imprinting a vortex in the condensate, one can generate robust topologically-protected embedded solitons, which may open up a way for the first experimental realization of 3D (matter-wave) embedded solitons as well as for monitoring the gap-soliton to embedded-soliton transition.
